# Disease control and functional outcome in three modern combined organ preserving regimens for locally advanced squamous cell carcinoma of the head and neck (SCCHN)

**DOI:** 10.1186/1748-717X-6-122

**Published:** 2011-09-23

**Authors:** Alexandra D Jensen, Jürgen Krauss, Wilko Weichert, Zazie P Bergmann, Kolja Freier, Jürgen Debus, Marc W Münter

**Affiliations:** 1 Dept of Radiation Oncology, INF 400, 69120 Heidelberg, Germany; 2 National Centre for Tumour Disease (NCT), INF 460, 69120 Heidelberg, Germany; 3 Institute of Pathology, INF 220/221, 69120 Heidelberg, Germany; 4 Dept of Head and Neck Surgery, INF 400, 69120 Heidelberg, Germany; 5 Dept of Oro-maxillofacial Surgery, INF 400, 69120 Heidelberg, Germany

## Abstract

**Purpose:**

To report our experience on disease control and functional outcome using three modern combined-modality approaches for definitive radiochemotherapy of locally advanced SCCHN with modern radiotherapy techniques: radiochemotherapy (RChT), radioimmunotherapy (RIT) with cetuximab, or induction chemotherapy with docetaxel, cisplatin, and 5-FU (TPF) combined with either RChT or RIT.

**Methods:**

Toxicity and outcome was retrospectively analysed in patients receiving definitive RChT, RIT, or induction chemotherapy followed by RChT or RIT between 2006 and 2009. Outcome was estimated using Kaplan-Meier analyses, toxicity was analysed according to CTCAE v 3.0.

**Results:**

Thirty-eight patients were treated with RChT, 38 patients with RIT, 16 patients received TPF followed by either RChT or RIT. Radiotherapy was mostly applied as IMRT (68%). Long-term toxicity was low, only one case of grad III dysphagia requiring oesophageal dilatation, no case of either xerostomia ≥ grade II or cervical plexopathy were observed. Median overall survival (OS) was 25.7 months (RChT) and 27.7 months (RIT), median locoregional progression-free survival (PFS) was not reached yet. Subgroup analysis showed no significant differences between TPF, RChT, and RIT despite higher age and co-morbidities in the RIT group. Results suggested improved OS, distant and overall PFS for the TPF regimen.

**Conclusion:**

Late radiation effects in our cohort are rare. No significant differences in outcome between RChT and RIT were observed. Adding TPF suggests improved progression-free and overall survival, impact of TPF on locoregional PFS was marginal, therefore radiotherapeutic options for intensification of local treatment should be explored.

## Introduction

The past decade has seen major changes in the clinical management of locally advanced squamous cell cancer of the head and neck (SCCHN). Concomitant cytostatic agents as well as major technical developments such as intensity-modulated radiotherapy (IMRT) and image-guided radiotherapy (IGRT) have changed standard practice. Concomitant platin-based radiochemotherapy has become one of the treatment standards [1-3]; however, improved outcome is bought at the cost of increased toxicity when compared to radiotherapy alone. Results comparable to concomitant radiochemotherapy were achieved by the introduction of targeted therapies: local control and overall survival rates were similar to historic controls in a large phase III trial comparing radioimmunotherapy with the monoclonal EGFR antibody cetuximab and radiation therapy only [4-6]. Interestingly, combined radioimmunotherapy with cetuximab did not show higher toxicity rates except for the typical acneiforme skin rash. This agent can therefore also be given to patients unable to tolerate the more toxic radiochemotherapy regimen. In the absence of direct or prospective randomised comparisons between the standard cisplatin regimen and cetuximab in concomitant chemoradiation, guidelines still recommend using standard regimen for patients fit enough to undergo chemotherapy [7]. Two recent trials evaluating taxane-based induction chemotherapy with docetaxel, cisplatin, and 5-FU (TPF) [8,9] have raised the interest in induction chemotherapy for SCCHN. Both trials resulted in an improvement of overall survival and progression-free survival. Although manageable, the TPF regimen is accompanied by sometimes marked toxicity and requires experienced management.

While addition of either concomitant or sequential chemotherapy regimen have been used to intensify radiotherapy, technical possibilities have also evolved within the past decade: intensity-modulated radiotherapy (IMRT) has rapidly been adopted as a therapeutic standard in the treatment of head and neck cancer due to high conformality and improved normal tissue sparing. In particular, salivary gland sparing leads to improved salivary gland function post radiotherapy and hence significant reduction of xerostomia as compared to conventional or three-dimensional techniques [10-13]. This has recently been verified in a prospective phase III trial comparing IMRT versus conventional techniques [14]. In a larger retrospective analysis, IMRT even lead to an improvement in overall survival as compared to standard techniques [15].

Neither of the three combined treatment modalities mentioned above have ever been directly compared in a clinical trial nor has the use of modern radiotherapy techniques in combination with these regimens ever been evaluated prospectively. Hence, clinicians need to rely on retrospective analyses and comparisons to evaluate potential routine use. Therefore, we report our experiences with the three regimens combined with IMRT techniques in our daily clinical practice.

### Patients and methods

Patients receiving definitive treatment for locally advanced SCCHN between 01/2006 and 06/2009 were identified retrospectively from our institutional database. Baseline characteristics as well as treatment parameters were retrieved from the hospital database in order to evaluate efficacy and outcome of the various regimens currently in use.

Only patients treated with a potentially curative intent were included in our analysis. All patients were staged prior to therapy with panendoscopy, CT of the head/neck and chest, abdominal ultrasound, and bone scan. Selection of specific combined regimen was based on the patients' overall performance status and co-morbidities in our institutional interdisciplinary head and neck tumour board. In cases of large tumour burden and good overall performance score (ECOG 0 or 1), induction chemotherapy was evaluated. Treatment standard at the time was combined chemoradiation according to the Staar protocol [16]. RIT was indicated in cases where concomitant chemoradiation was prohibitive due to poor overall performance status (ECOG 2) or multiple co-morbidities.

#### Radiation therapy: immobilisation/planning procedures

Patients were immobilized using either a combination of individual scotch-cast mask and vacuum pillow or individual thermoplastic head masks incl. shoulder fixation (HeadStep^®^, ITV). Planning examinations included CT-scan and contrast enhanced MRI for 3D image correlation. Treatment isocentres were localised stereotactically until 2008 and under image guidance (virtual simulation) from 2008.

#### Radiation therapy: treatment

Target volumes were delineated in accordance with current guidelines and recommendations [17-19]. RT was prescribed to 66 - 72 Gy to the primary tumour/involved nodes and between 54 - 57.6 Gy to the bilateral neck. Intensity-modulated radiation therapy (IMRT) is the treatment of choice for all patients, 3D and conventional techniques were only used in patients unable to tolerate longer treatment times (IMRT: approx. 15-20 min) per fraction. Only patients in severely reduced performance state received 2D radiation, therefore use of the classical concomitant boost concept was prohibitive. The median dose to the contralateral parotid was below 27 Gy; if possible, also the ipsilateral parotid gland was spared. Maximum doses to the spinal cord were limited to < 40 Gy. IMRT treatment was either carried out at a 6 MV linac in step-and-shoot technique or at a 6 MV tomotherapy unit.

Patient position was verified at least weekly using CT-control scans, MV cone-beam CT, and portal images.

#### Radiochemotherapy with carboplatin/5-FU

According to our institutional protocols, patients with locally advanced SCCHN are treated with carboplatin and 5-FU according to the protocol published by Staar et al. [16].

Carboplatin is given at 70 mg/m^2 ^as one-hour infusion and 5-FU as 600 mg/m^2 ^(23 h) on days 1 to 5 and days 29 to 33 of radiation. Patients are provided with standard antiemetic prophylaxis and hydration.

#### Radioimmunotherapy with cetuximab

After administration of anti-histamines and corticosteroids, the monoclonal antibody cetuximab (Erbitux^®^) was administered at 400 mg/m^2 ^body surface loading dose seven days prior to RT-treatment start. Weekly administrations of cetuximab at 250 mg/m^2 ^body surface followed for the duration of radiotherapy according to general recommendations of the vendor.

### Induction chemotherapy with TPF

Patients received induction chemotherapy with docetaxel/cisplatin/5-FU (TPF) according to the schedule described by Vermorken et al. [9] and to standard recommendations of the vendors. In addition, patients received prophylactic antibiotic treatment, usually consisting of ciprofloxacine 500 mg po bid for 10 days starting one week after commencement of each cycle. G-CSFs were administered as indicated. The first re-staging was carried out after the second cycle by either CT or MRI.

#### Analysis

Treatment response was analysed 6 weeks post completion of radiotherapy (1^st ^f/u) and 3 months thereafter (2^nd ^f/u) based on available diagnostic imaging (CT or MRI) and clinical examinations according to RECIST criteria [20]. Treatment outcome/survival rates were evaluated using higher non-parametric statistics (Kaplan-Meier survival analysis [21]/log-rank and Wilcoxon test). Progression-free survival was defined as the time from start of radiation therapy until first event (i.e. locoregional relapse, distant metastases, death). Similarly, overall survival was calculated from start of radiotherapy until death from any cause. To test for differences between the treatment groups, a student-t test or Fisher's exact-test were used where appropriate. Statistics were carried out using the Addinsoft xlstat life 2011^© ^software package. Toxicity was evaluated based on recorded clinical examinations and documentation within the individual patient charts and assessed using NCI CTC v 3.0.

## Results

After exclusion of patients with nasal or paranasal sinus cancers, partial resections, and re-irradiations, 76 patients receiving primary radiotherapy for locally advanced carcinoma of the head and neck between 01/2006 and 06/2009 were identified. Thirty-eight patients received primary radiochemotherapy (RChT), 38 patients radioimmunotherapy (RIT) with the EGFR-inhibitor cetuximab. The cohort included 16 patients receiving upfront induction chemotherapy with docetaxel, cisplatin, and 5-FU (TPF) before either radiochemotherapy (10 pts) or radioimmunotherapy (6 pts).

Patient baseline characteristics, treatment sites, and stages are displayed in table 1. All patients received irradiation to bilateral nodal levels (levels II-V), most patients were treated by IMRT (30/38 pts in the RChT group, 22/38 pts in the RIT group), median doses to the primary and involved nodes were 68.2 Gy (RChT) and 66 Gy (RIT) (table 2). Thirty-seven out of 38 patients in the RChT group and 34/38 patients in the RIT group completed their treatment as scheduled. One patient in the RChT group discontinued therapy due to progressive renal failure, three patients in the RIT group declined further therapy. Thirty-four patients have deceased as of January 2011. Median follow-up in the RChT cohort is 18.7 months [0.63 - 51.3 months] compared with 10.8 months [0.2 - 76.2] in the RIT cohort.

**Table 1:** Patient baseline characteristics and demographics

		radiochemotherapy	radioimmunotherapy	TPF induction
**site**		**pts, (%)**		
	hypopharynx	17 (44.7)	3 (7.9)	5 (31.3)
	oropharynx	7 (18.4)	6 (15.8)	3 (18.8)
	base of tongur	5 (13.2)	4 (10.5)	1 (6.3)
	oral cavity	4 (10.5)	12 (31.6)	1 (6.3)
	oro-/hypopharynx	3 (7.9)	5 (13.2)	3 (18.8)
	larynx	1 (2.6)	7 (18.4)	1 (6.3)
	oropharynx+hypopharynx	1 (2.6)		1 (6.3)
	lip		1 (2.6)	
				
				
				
**stage**	T1	1 (2.6)	6 (15.8)	
	T2	3 (7.9)	6 (15.8)	1 (6.3)
	T3	4 (10.5)	4 (10.5)	2 (12.5)
	T4	30 (78.9)	22 (57.9)	13 (81.3)
				
	N0	3 (7.9)	10 (26.3)	1 (6.3)
	N1	2 (5.3)	4 (10.5)	2 (12.5)
	N2a		3 (7.9)	
	N2b	5 (13.2)	6 (15.8)	4 (25)
	N2c	23 (60.5)	15 (39.5)	8 (50)
	N3	4 (10.5)		1 (6.3)
	pN2b	1 (2.6)		
				
	M1	2 (5.3)	4 (10.5)	0
				
	primary treatment	35 (92.1)	26 (68.4)	15 (93.8)
	local relapse	3 (7.9)	12 (31.6)	1 (6.3)
	seconday tumours	2 (5.3)	2 (5.3)	2 (12.5)
				
Total		38	38	16
				
**age [a]**	median	61.1	71.4	60.3
	range	17 - 74	50 - 85	17 - 68
		p < 0.001		p < 0.001

**Table 2:** Treatment characteristics

		radiochemotherapy	radioimmunotherapy
**treatment**		**pts**	
	primary radiochemotherapy	28	32
	TPF induction followed by RChT	10	6
			
	IMRT	30	22
	3D	2	7
	2D	6	9
			
	regular completion of treatment	37	34
			
**dose (median; [Gy])**	primary/involved nodes	68.2	66
	range	22 - 72	16 - 70.6
			
	neck	56	55.8
	range	49.8 - 60	16 - 60

**Figure 1:**
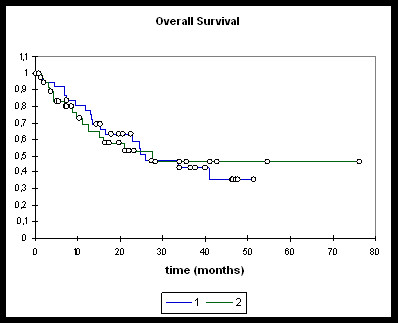
**Overall survival**. **1: **RChT; **2: **RIT. RChT (n = 38), RIT (n = 38). RChT OS @ 2 years: 58.9%. RIT OS @ 2 years: 52.7%. Log-rank/Wilcoxon ns.

**Figure 2:**
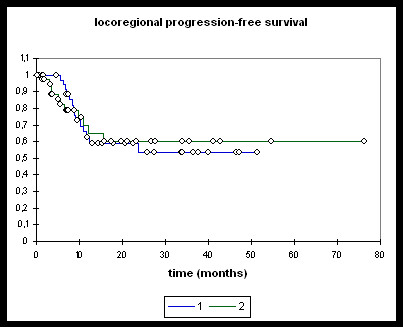
**Locoregional progression-free survival**. **1: **RChT; **2: **RIT. RChT (n = 38), RIT (n = 38). RChT @ 2 years: 53.7%. RIT @ 2 years: 60.0%. Log-rank/Wilcoxon ns.

Median overall survival (Figure 1) in the entire RChT cohort (n = 38) was 25.7 months and 27.7 months in the entire RIT group (n = 38). Median locoregional progression-free survival (Figure 2) was not reached in either of the groups. Neither overall nor locoregional progression-free survival differed significantly between the two groups.

**Figure 3:**
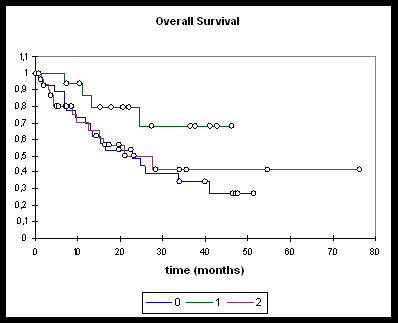
**Overall survival**. **0: **RChT; **1: **TPF; **2: **RIT. RChT median: 22.8 months [95%-CI: 13.1 - 40.9]; OS @ 2 years: 48.7%. TPF median: not reached; OS @ 2 years: 79.3%. RIT median: 27.7 months [95%-CI: 12.6 - 33.9]; OS @ 2 years: 50%. RChT vs TPF: logrank 0.05, Wilcoxon 0.067. RChT vs RIT: ns. RIT vs TPF: ns.

**Figure 4:**
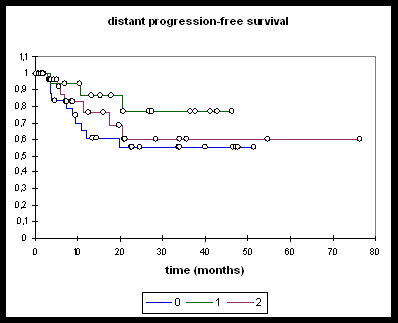
**Distant PFS**. **0: **RChT; **1: **TPF; **2: **RIT. RChT: @ 2 years: 55.1%. TPF: @ 2 years: 76.9%. RIT: @ 2 years: 60.2%. RChT vs TPF, RChT vs RIT, RIT vs TPF: ns.

**Figure 5:**
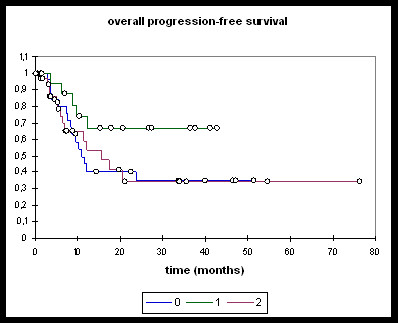
**Overall PFS**. **0: **RChT; **1: **TPF; **2: **RIT. RChT median: 10.9 months [95%-CI: 8.3 - 33.7]; PFS @ 2 years: 34.7%. TPF: PFS @ 2 years: 66.6%. RIT median: 15.6 months [95%-CI: 7.1 - 33.9]; PFS @ 2a: 34.5%. RChT vs TPF, RChT vs RIT, RIT vs TPF: ns.

**Figure 6:**
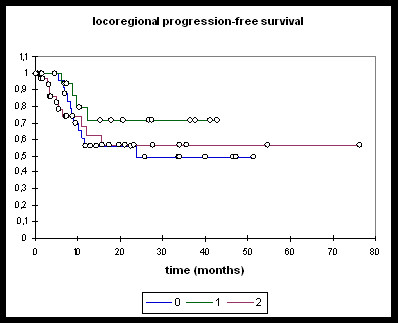
**Locoregional PFS**. **0: **RChT; **1: **TPF; **2: **RIT. RChT median 23.8 months [95%-CI: 10.2 - 33.7]; locoregional PFS @ 2 years: 49.0%. TPF: @ 2 years: 71.4%. RIT: @ 2 years: 56.7%. RChT vs TPF, RChT vs RIT, RIT vs TPF: ns.

Patients treated with induction chemotherapy followed by either RChT or RIT (n = 16), RChT (n = 28), and RIT (n = 32) were analysed separately (Figures 3, 4, 5, 6).

Overall and progression-free survival again was not significantly different between the three treatment groups although the TPF-group was trending towards improved overall survival (Figure 3), distant PFS (Figure 4) and overall PFS (Figure 5). No significant differences could be observed regarding locoregional PFS (Figure 6). Radiochemotherapy and radioimmunotherapy following TPF induction were compared but neither survival nor local control differed significantly (data not shown) therefore, analysis within one group seems justified.

Toxicity was within the expected range (table 3), mucositis being the most common side-effect and gradually resolving up to the first follow-up 6 weeks post treatment. 48 patients had a PEG tube insertion, 4 patients a central venous catheter. Acute dysphagia rates were 35.5% (27 pts, RChT) and 32.9% (25 pts, RIT).

**Table 3:** Toxicity

toxicity	CTC grade (if applicable)	end of treatment		1st f/u		2nd f/u	
		**RChT**	***RIT***	**RChT**	***RIT***	**RChT**	***RIT***
**dysphagia**	I	1	*1*	5	*5*	1	
	II	11	*16*	7	*8*		
	III	15	*8*	1	*2*	1	
							
**mucositis**	I	1	*1*	3	*1*		
	II	20	*19*	1			
	III	3	*8*				
						9	*2*
**xerostomia**	I	4		7	*11*	1	*1*
	II						
							
**laryngeal oedema**		1				2	
**lymph oedema**				1	*1*	3	
							
							
**weight loss (median, kg)**		4	*2*	p = 0.01			
**range (kg)**		0 - 14	*0 - 9*				
**PEG (pts)**		27	*21*				
**CVK (pts)**		3	*1*				
**toxicity**	**CTC grade (if applicable)**	**end of treatment**		**1st f/u**		**2nd f/u**	
		**RChT**	***RIT***	**RChT**	***RIT***	**RChT**	***RIT***
**dysphagia**	I	1	*1*	5	*5*	1	
	II	11	*16*	7	*8*		
	III	15	*8*	1	*2*	1	
							
**mucositis**	I	1	*1*	3	*1*		
	II	20	*19*	1			
	III	3	*8*				
						9	*2*
**xerostomia**	I	4		7	*11*	1	*1*
	II						
							
**laryngeal oedema**		1				2	
**lymph oedema**				1	*1*	3	
							
							
**weight loss (median, kg)**		4	*2*	p = 0.01			
**range (kg)**		0 - 14	*0 - 9*				
**PEG (pts)**		27	*21*				
**CVK (pts)**		3	*1*				

Dysphagia gradually resolved with time, late swallowing dysfunction was observed in 2 patients in the RChT group, one of them needing oesophageal dilatation 2 years post completion of treatment. Two patients in the RChT group developed aspiration pneumonia.

Although 9 patients a priori had impaired salivary gland function documented by salivary flow scintigraphy before treatment start, only 2 patients complained of mild xerostomia beyond the 2^nd ^follow-up. There was no case of CTC grade II xerostomia. Despite high radiation doses given in the proximity of the brachial plexus due to cervical lymph node metastases, no cervical plexopathy was observed.

Six of 76 patients underwent salvage surgery for locoregional relapse, 3 patients were treated by laryngectomy (RChT: 2 pts, RIT 1 pt), 1 patient by partial glossectomy (RChT) and 3 patients (RChT) received salvage neck dissection, therefore the overall organ preservation rate in this cohort is 96.1%.

## Discussion

Side-effects of the two principal treatment regimens RChT and RIT were within the expected range. As expected, acute side-effects were slightly higher in the chemoradiation group though not statistically significant. Overall late toxicities were mild: we observed only one case of CTC grade III late dysphagia requiring treatment by upper oesophageal dilatation. Following this procedure, the patient patient's symptoms have resolved. No other higher-grade (CTC ≥°II) swallowing dysfunction was recorded. However, one of the major limitations in the evaluation of swallowing dysfunction in our patients is the fact that swallowing function was not evaluated prospectively. Therefore initial swallowing function tests are not available for most of the patients. Evaluation was carried out on documented ENT follow-up examinations, which mostly consisted of clinical and endoscopical examination post therapy. Hence the rate of silent aspirations in our patients may presumably be higher than the actual rates reported here [22-25]. Nevertheless, most patients did not experience complications nor have they subjectively reported swallowing problems. Regarding the initially very advanced tumour stages, this encouraging fact may be attributed to our routine use of IMRT [24,25].

Survival as well as control rates are consistent with results from previously reported trials [16,4,26-30]. Locoregional control at one year between 49% (RChT) and 56.7% (RIT) was slightly lower than outcome reported in the RTOG 99-14 trial [27] using altered fractionation in addition to chemoradiation but comparable to the results reported by Staar et al [16]. The results for local control rates are also in the range reported by Bonner et al [4,5] despite the high rates of T4 tumours both in our RChT and RIT group and despite the selection bias in the RIT group. All our RIT patients had a very limited to poor pre-therapeutic performance status and most were also elderly patients. Demonstrated by Cooper et al [31] and more recently by Agarwal and Siddiqui et al. [32,33] one of the most important predictive factors for patient outcome are tumour and nodal stage as well as initial performance status [34] and advanced age. Hence, our radioimmunotherapy patients in fact represent a very negative pre-selection with a combination of adverse prognostic factors not represented in prospective and/or randomised clinical trials. In the Bonner trial [4], such patients were shown to benefit less than younger, fitter patients.

Even more remarkable is the fact that neither survival nor locoregional control rates differed significantly between radiochemotherapy and and radioimmunotherapy in our cohorts further supporting data from retrospective comparisons by Caudell et al. [4,6]. Overall survival rates (at 2 years: 48.7% (RChT), 79.3% (TPF), 50.0% (RIT) are even slightly higher than in our reference trial [16] despite the fact that also older patients and patients with limited pulmonary metastases were included in our analysis. In addition, more patients in the RIT group discontinued treatment due to worsening of overall condition.

For overall progression-free survival, distant progression-free survival, and overall survival, TPF induction showed a visible trend towards improved outcome independent of the subsequent treatment modality (RChT or RIT), although this trend did not reach statistical significance. This is in line with a randomized phase III trial presented by Hitt et al at at ASCO 2009 [35]. It cannot be excluded that age and performance state of the patients receiving TPF has caused parts of the improvement in progression-free and overall survival. While this may be true, our data also shows that improvement of survival rates beyond the results reported in the large radiochemotherapy trials is possible in carefully selected patients and can hence be an option in the hands of experienced oncologists.

The benefit in locoregional progression-free survival - though not statistically significant - seems to be only slightly higher for the TPF group. Also patient numbers are small, this may suggest induction chemotherapy with TPF influences progression-free survival selectively by reduction of distant metastases rather than improvement of local control, which is supported by the TAX 323 and 324 trials [8,9]. Further analysis of our patients treated with TPF followed by radiochemotherapy or radioimmunotherapy revealed no significant difference between RChT and RIT following TPF. The number of patients treated with this regimen at our institution though, is still small. So far, TPF followed by RIT could be favoured due to lower accompanying toxicity and potentially higher patient compliance. The TREMPLIN trial addresses this question by randomizing between chemoradiation and radioimmunotherapy following induction chemotherapy. Until final results of this trial are available however, this question cannot be reliably answered [36].

Long-term local control remains an issue in the age of concomitant radiation schedules, targeted therapies and induction chemotherapy. TPF was not overtly successful with respect to improvement in local control in our cohort. In addition, radiochemotherapy and radioimmunotherapy do not show significant differences in local control rates either in the study published by Caudell [6] or in our patients. One potential treatment regimen, which might prove beneficial in this context, is the combination of platin-based radiochemotherapy and immunotherapy currently under evaluation in the RTOG 0522 [37] and also in our own REACH trial [38]. However, improvement by combination of these agents may have limited effects and will only potentially be feasible if toxicity rates are not significantly increased. Since RChT and RIT show similar results with respect to local control rates, further intensification of radiotherapy needs to be explored to improve local control. De-escalation of the systemic components (e.g. cetuximab only) may be feasible especially in regimens including TPF induction and improve patient compliance. Most of our patients did receive IMRT as simultaneous integrated boost, is there still room to intensify radiotherapy by altered fractionation in the age of IMRT? The REACH trial [38] will try to address this issue by investigating the combination of radiochemoimmunotherapy with carboplatin, 5-FU, and cetuximab and intensity-modulated radiation therapy as concomitant boost. In addition, particle therapy will shortly become routinely available in some centres and offers the benefit of extremely high conformality and increased relative biological effectiveness. Therefore, integration of this technique into standard radiochemo/radioimmunotherapy regimens might result in higher control rates. Potential gains warrant further exploration of this hypothesis but need to be explored in active clinical trials such as the TPF-C-HIT trial [39].

## Conclusion

Even in patients with a compromised performance status, modern combined-modality regimens lead to local control and survival at rates comparable to large landmark trials. Observed acute and late toxicity rates are low: persistent swallowing dysfunction is rare in our cohort, no cervical plexopathies were observed.

No significant difference between radiochemotherapy with carboplatin/5-FU and radioimmuntherapy with cetuximab was observed. TPF induction therapy was associated with improved progression-free as well as overall survival. However, impact of TPF on locoregional progression-free survival was only marginally positive and not statistically significant, therefore novel radiotherapeutic options for intensification of local treatment should be explored.

## Conflict of interest

JD is a member of Merck KGa advisory board, all other authors declare no competing interests.

## Authors' contributions

All authors read and approved the final manuscript
